# The Possible Emergence of Life and Differentiation of a Shallow Biosphere on Irradiated Icy Worlds: The Example of Europa

**DOI:** 10.1089/ast.2016.1600

**Published:** 2017-12-01

**Authors:** Michael J. Russell, Alison E. Murray, Kevin P. Hand

**Affiliations:** ^1^ Jet Propulsion Laboratory, California Institute of Technology, Pasadena, California.; ^2^ Division of Earth and Ecosystem Sciences, Desert Research Institute, Reno, Nevada.

## Abstract

Irradiated ice-covered ocean worlds with rocky mafic mantles may provide the conditions needed to drive the emergence and maintenance of life. Alkaline hydrothermal springs—relieving the geophysical, thermal, and chemical disequilibria between oceans and tidally stressed crusts—could generate inorganic barriers to the otherwise uncontrolled and kinetically disfavored oxidation of hydrothermal hydrogen and methane. Ionic gradients imposed across these inorganic barriers, comprising iron oxyhydroxides and sulfides, could drive the hydrogenation of carbon dioxide and the oxidation of methane through thermodynamically favorable metabolic pathways leading to early life-forms. In such chemostatic environments, fuels may eventually outweigh oxidants. Ice-covered oceans are primarily heated from below, creating convection that could transport putative microbial cells and cellular cooperatives upward to congregate beneath an ice shell, potentially giving rise to a highly focused shallow biosphere. It is here where electron acceptors, ultimately derived from the irradiated surface, could be delivered to such life-forms through exchange with the icy surface. Such zones would act as “electron disposal units” for the biosphere, and occupants might be transferred toward the surface by buoyant diapirs and even entrained into plumes. Key Words: Biofilms—Europa—Extraterrestrial life—Hydrothermal systems. Astrobiology 17, 1265–1273.

## 1. Introduction

On Earth, the geochemical utility of life can, in part, be simplified to the hydrogenation of carbon dioxide. Such hydrogenations yield an ever-renewed stock of highly specified organic molecules—the so-called CHNOPS with a typical bonding motif -C–C(H_2_)–N(H)–C–O (Bernal, 1960; Lassiter, 1986; Fuchs, 1989, 2011). However, the significance of the proton and electron flux must also be appreciated, since those processes are at the root of life's role in free energy transfer and transformation (Mitchell, 1961; Szent-Györgyi, 1968). Viewed in these terms, life is a mechanism that hastens the flow of “hot” electrons to available electron acceptors (*e.g.,* Steele, 2003; Russell *et al.,* 2003, 2014; Nitschke and Russell, 2011). Here, we suggest that life may have emerged on irradiated icy worlds such as Europa, in part as a result of the chemistry available within the ice shell, and that it may be sustained still, immediately beneath that shell.

## 2. The Drive to Life on Ice-Covered Ocean Worlds with Rocky Mantles

**FIG. 1.**
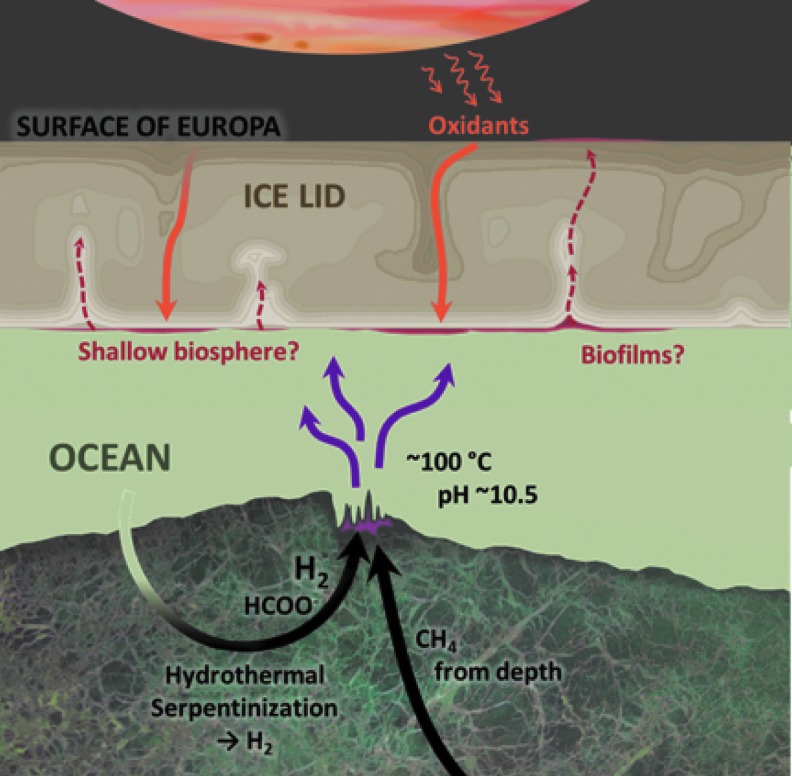
Model for the emergence of life on Europa at an alkaline hydrothermal mound (Russell *et al.,* 2014, and see Vance *et al.,* 2016). Also indicated is a hypothesized rapid migration of microbes and nanobes entrained within buoyant thermal plumes toward oxidant-rich areas at the base of the ice lid. These areas act as “electron disposal units” and are derived through subduction of oxidants from the exterior produced by high-energy electron radiation from Jupiter (Bolton *et al.,* 2002). In turn, portions of this shallow and buoyant biosphere may be returned to the surface through ice tectonics or sucked into the source regions of water vapor/ice jets on, for example, Europa and Enceladus (Squyres and Croft, 1986; Roth *et al.,* 2014; Lorenz, 2016; Sparks *et al.,* 2016; and McKay *et al.,* 2008). Along with the standard methods for flying through any existing plumes and analyzing the surface, future missions could employ “Under-Ice Buoyant Rovers for Exploration” of the putative biosphere (Berisford *et al.,* 2012, and see Ananthaswamy, 2013). The ice shell is partly based on Showman and Han (2005) and Kalousová *et al.* (2014). Not to scale.

In cases where ice-covered ocean worlds with rocky cores are subject to surface irradiation and tidal forces, such systems may reach a metastable dynamic state. In these systems, electron-bearing fuels will be produced in serpentinization (rock-water) reactions as salty ocean waters are reduced to hydrogen and formate on gravitation into the primitive ultramafic crust (Windman *et al.,* 2007; Russell *et al.,* 2010; Vance *et al.,* 2016). Primeval (abiotic) methane could be leached concomitantly from the same source (*e.g.,* Watanabe *et al.,* 1983; Sherwood Lollar *et al.,* 2007; McCollom and Donaldson, 2016). Likewise, hydrogen and methane could be entrained convectively to the ocean through widespread and continuous submarine alkaline hydrothermal activity (Hand *et al.,* 2007; Vance *et al.,* 2007, 2016; Vance and Goodman, 2009; Travis *et al.,* 2012; Russell *et al.,* 2014) (Fig. 1). Along with methane, the hydrothermal fluids feeding the springs could also have entrained vital nutrients in the form of further reduced volatiles to be expected on a moon such as Europa with a mantle and crust dominated by fayalite (ferrous iron-rich olivine), that is, carbon monoxide, ammonia, and hydrogen sulfide (Fig. 1) (Anderson *et al.,* 1998; Sohl *et al.,* 2002; McDermott *et al.,* 2015; and see Wood *et al.,* 2006; Gaillard *et al.,* 2015). Other entities could be leached into the same hydrothermal fluids, such as the all-important phosphorous (perhaps from dissolution of phosphide) along with the trace metals, including molybdenum, required of all Earthly life (Pasek *et al.,* 2011; Schoepp-Cothenet *et al.,* 2012; Russell *et al.,* 2014; Pasek, 2016).

On Earth, hydrogen, or more precisely its component electrons and protons, is life's must-have fuel. And, given a supply of carbon dioxide and other, higher potential oxidants such as nitrate, nitrite, sulfite, sulfur, and ferric iron in early mildly acidic oceans, life may have been forced into being in order to resolve these electrogeochemical disequilibria. The best candidate for such interactions would be hydrothermal mounds and sediments comprising ferrous-ferric oxyhydroxides and sulfides precipitated where alkaline hydrothermal fluids interfaced acidulous ocean water (Russell *et al.,* 1994, 2003, 2014; Pasek and Greenberg, 2012; Tosca *et al.,* 2016; Halevy and Bachan, 2017). Such mounds could have functioned as long-lasting, stably operating electrogeochemical reactors (Russell, 2007). The vectorial proton and redox gradients obtaining at, and near, the surface of the mounds are comparable to those that “energize” life as we know it on Earth (Kelley *et al.,* 2001; Martin *et al.,* 2008; Branscomb and Russell, 2013; Herschy *et al.,* 2014; Branscomb *et al.,* 2017). Moreover, the transition metals required for catalysis—and which are affine with the active centers of metalloenzymes—would also be available in the oxyhydroxides and sulfides constituting the mounds (Nitschke *et al.,* 2013).

It is these physicochemical disequilibria that are, in the alkaline hydrothermal vent model, proposed to be the founding drivers of biogenesis needed to reduce CO_2_ to simple organic acids, alcohols, and hydrocarbons, to fix nitrogen and to drive other required first-step endergonic reactions that eventuated in cellular life (Russell *et al.,* 2003; Martin and Russell, 2007). We see such worlds as batteries with outputs approaching a volt or so (Russell and Hall, 1997), that drive prokaryotic cells with the same spatiality as natural fuel cells (Mitchell, 1967; Russell, 2007). And the specific iron-bearing minerals dosed with nickel and molybdenum—mainly green rust and lesser mackinawite and greigite—that make up such inorganic layers and micro-conduits constituting the metal-bearing electrodes of the precipitate mound have, it is proposed, the capability to couple the proton and redox gradients to carbon and nitrogen fixation (Génin *et al.,* 2005, 2006, 2008; Nitschke *et al.,* 2013; Barge *et al.,* 2014, 2015a, 2015b; Russell *et al.,* 2014; White *et al.,* 2015; Branscomb *et al.,* 2017; Halevy *et al.,* 2017; *cf.* Mloszewska *et al.,* 2012; Peacock *et al.,* 2016).

## 3. The Ensuing Biosphere

**FIG. 2.**
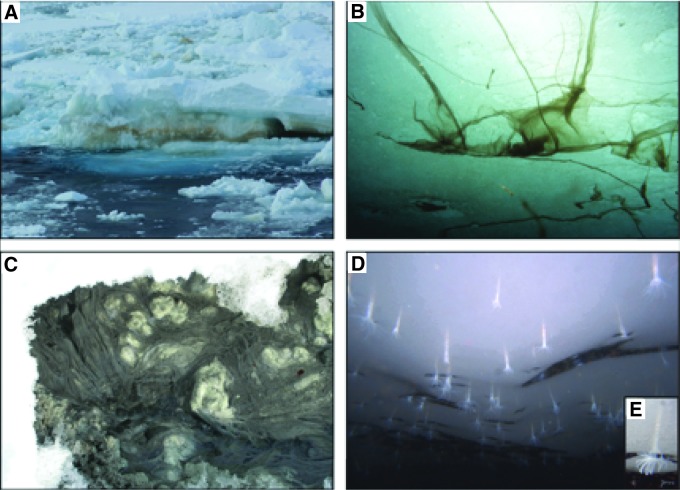
Ice-water interfaces support dense communities of microscopic and even macroscopic life on Earth. (**A**) Dense multispecies communities of algae, bacteria, heterotrophic protists, and even multicellular invertebrates form annually under spring sea-ice conditions in the Southern Ocean when ample sunlight fuels photosynthesis of ice algae. The orange-brown carotenoid pigments in dense diatom aggregations in sea ice are visible to the eye in the image taken from the Northern Antarctic Peninsula in August (photo source: A.E. Murray). (**B**) Spring conditions in the Arctic also support large, macroscopic filamentous forms of the ice-associated diatom, *Melosira arctica,* shown attached to the underside of the sea ice. (© AWI/Gutt, doi:10.1594/PANGAEA.820720). (**C**) Runoff from Gypsum Springs, a sulfurous spring on Axel Heiberg Island, in the Canadian High Arctic, is home to filamentous sulfur-oxidizing bacterial streamers that underlie the snow and ice cover, site GH-4 (water temperature ∼5–6.9°C; image is ∼30 cm in diameter; Niederberger *et al.,* 2009). (**D**) The underside of the Ross Ice Shelf has recently been found to harbor fields of the newly described ice-dwelling sea anemone *Edwardsiella andrillae,* which lives embedded in the ice. **(E)** Inset shows a close-up image of the under ice-embedded anemone, *Edwardsiella andrillae*. Diameter across tentacles is ∼1–2 cm. The image was captured using the ROV SCINI at Coulman High site (adopted from Murray *et al.,* 2016). The under-ice-shelf ecosystems on Earth though potentially sustained by fuels sourced from the open ocean may be relevant proxies for ice shells of outer Solar System ocean worlds. (Vick-Majors *et al.,* 2016).

In comparing active icy worlds such as Europa and Enceladus to our own planet, major differences must be taken into account (Pappalardo *et al.,* 1998; Sotin *et al.,* 2002; Waite *et al.,* 2006, 2017; Nimmo *et al.,* 2007; McKay *et al.,* 2008; Schmidt *et al.,* 2011; Glein *et al.,* 2015). On Earth, the ocean is mostly solar heated. And ice-water associated interfaces are known to support dense congregations of life as a result of (i) Sun-derived energy infiltrating nutrient-bathed sea ice habitats (Fig. 2A, 2B), (ii) dissolved, reduced sulfur sources that drive chemoautotrophic biofilms in Arctic terrestrial cold-seep formations (Fig. 2C), or (iii) putative detrital organic matter supplies fueling under-ice-shelf ecosystems that even harbor multicellular life (Fig. 2D). On icy worlds, however, the oceans are heated from below, and the ice shells will confer upon the sub-ice ocean the “steady state” characteristics of a giant natural chemostat (Goodman *et al.,* 2004; Glein *et al.,* 2015). Under such conditions, organisms and biological processes could be limited by the availability of oxidants, that is, the compounds needed to “breathe” (Nealson *et al.,* 2002). Iron and manganese oxides and oxyhydroxides, sulfuric acid, sulfates, sulfur, carbonates, and even oxygen itself are possible candidate electron acceptors produced and concentrated on the heavily irradiated surface of certain ice shells (Chyba and Hand, 2001; McCord *et al.,* 2001; Carlson *et al.,* 2002; Zolotov and Shock, 2003, 2004; Hand *et al.,* 2007; Vance *et al.,* 2014, 2016; and see Nealson *et al.,* 2002; Lin *et al.,* 2006; Milucka *et al.,* 2012; Egger *et al.,* 2017).

Hydrogen peroxide produced in the purest ice regions via radiolysis could subsequently react with hydrogen sulfide and ammonia in the ice, yielding oxidants (*e.g.,* sulfite, nitrate, and nitrite) that are potentially more amenable as acceptors along metabolic pathways than just CO_2_ or carbonate (Hand and Brown, 2013; Loeffler and Hudson, 2015; *cf.* Zolotov and Shock, 2004; Wong *et al.,* 2017). Early in Europa's history, radiolytically generated oxidized entities may have been subducted toward the ocean through occasional overturn of the ice shell, perhaps such that it produced an initially acidic ocean (Pappalardo *et al.,* 1998; Bolton *et al.,* 2002; Hand *et al.,* 2006; Greenberg, 2010; Pasek and Greenberg, 2012; Kattenhorn and Prockter, 2014; Vance *et al.,* 2016). It is under such conditions that proton and oxidation/reduction gradients imposed across precipitate mounds at submarine alkaline springs (electrons attracted outward, protons in toward the alkaline interior) could potentially have been harnessed through the emergence of metabolism and thereby life, much as thermal gradients can be harnessed to drive the onset of convection (Russell *et al.,* 2003). As one example, given the potentially large flux of radiolytic sulfate, Zolotov and Shock (2003) calculated that sulfate is a strong enough oxidant to accept electrons from both hydrogen and methane, which could then drive the necessary redox gradient across hydrothermal systems at Europa's seafloor. Nitrate and/or nitrite are even more attractive electron acceptors, as they may be reduced to ammonium in such circumstances, which could be employed to augment the amination of the carboxylic acids (Raulin, 2005; Flores *et al.,* 2016; Russell and Nitschke, 2017). Carbonate (or CO_2_) is a lower potential acceptor but has the advantage of also providing further carbon through a variant of the acetyl coenzyme-A pathway (Russell and Nitschke, 2017).

Fed by what may have been a plenitude of reductants (electron donors such as H_2_, CH_4_, and ferrous iron) from hydrothermal activity, and an ocean with dissolved oxidants providing a geochemical gradient, the first life-forms could potentially follow some of the same metabolic pathways known to be at, or near, the root of the earliest life on Earth, that is, acetogenesis and methanotrophy (Russell and Martin, 2004; Nitschke and Russell, 2013; Russell and Nitschke, 2017; *cf.* Mikucki *et al.,* 2009; Waite *et al.,* 2017). If, during periods of slowed or stalled ice-ocean exchange, SO_4_^2-^ and CO_2_ and other electron acceptors were to be rapidly drawn down by participants in the biofilm, the oxidant-limiting conditions would be created and an “oxidant crisis” ensue. Models for transport of material within Europa's ocean indicate that hydrothermal plumes could be well constrained within the ocean (primarily by the Coriolis force and thermal gradients), leading to effective delivery through the ocean to the ice-water interface (Goodman *et al.,* 2004; Goodman and Lenferink, 2012; Travis *et al.,* 2012; Goodman, 2016). Organisms fortuitously transported from hydrothermal systems to the ice-water interface along with unspent fuels could potentially access a larger abundance of oxidants directly from the ice (Fig. 1) (*e.g.,* Raymond *et al.,* 2008). Importantly, oxidants might only be available where the ice surface has been driven to the base of the ice shell (*e.g.,* Showman and Han, 2005). These sites could be geographically separated from upwelling sites within the ice shell driven by heat from hydrothermalism. Opportunistic life-forms could rely on lateral transport (*e.g.,* ocean currents) along the ice-water interface to reach sites of down-welling convection in the ice that might bring radiolytically produced oxidants to the ocean (Fig. 1).

In such chemostatic conditions, a coevolutionary mutualistic drive toward lowering the minimal requirement for electron acceptors while recycling electrons would be expected, homologous to dynamic growth in a Winogradsky column (*e.g.,* Schlegel and Jannasch, 2006; Castelle *et al.,* 2015; *cf.* Fernández *et al.,* 1999; Rinke *et al.,* 2013). This ocean-deep chemostat presents a rather strange and challenging situation in which the long-term supply of useful hot electrons on the one hand, and of oxidants on the other, would be dramatically separated spatially by the ∼100 km deep ocean desert. Yet carbon as abiotic CH_4_ and formate exhaled hydrothermally from the crust, as well as in the form of CO_2_ from melting of the ice shell, could be continually supplied to autotrophic metabolic life-forms. Heterotrophic activity could recycle much of the autotrophic waste and detritus and contribute to a thickening of the ice-bound biosphere, undiluted by sediment—a contrast to our own deep biosphere (Kepner *et al.,*^1998^; López-Bueno *et al.,* 2009; Rinke *et al.,* 2013). In these chemostatic conditions, populations could grow asymptotically to a stable, though dynamic, equilibrium (Fernández *et al.,* 1999; De Roos, 2004; Nealson *et al.,* 2005).

Such an evolutionary drive for speciation and potential diversity of metabolisms could create an efficient and durable ecosystem that would continue to draw down nutrients by maximizing the overall cellular electron-to-nutrient flux, thus simultaneously increasing a microbe's own waste, to be absorbed as nutrients by their adaptable and less discriminating heterotrophic neighbors (*cf.* Braakman *et al.,* 2017; and see Fernández *et al.,* 1999; Rinke *et al.,* 2013). Typical, rather insoluble, trace elements could also be recycled in organic chelates (*cf.* Milner-White and Russell, 2008). Thus, a strong effect of electron uptake would be to minimize other waste products through recycling by emerging heterotrophic, fermenting, and symbiotic microbes, and nanobes (Uwins *et al.,* 1998; Rinke *et al.,* 2013; Kuhn *et al.*, 2014; Luef *et al.,* 2015; *cf.* Stolz, 2017).

The overall effect of this process would be to strongly concentrate any putative cells by many orders of magnitude at these sites. This could be a critical consideration for life detection and the finding of potential biosignatures within Europa's ice (Hand *et al.,* 2017). An active ice-water interface could reach cell densities of 10^5^ to 10^8^ cells mL^−1^, comparable to microbial mats or other chemically rich interfaces on Earth (Nealson *et al.,* 2005; Hand *et al.,* 2009) (Fig. 2C). This is considerably higher than cell densities found in the accretion ice of subglacial Lake Vostok in Antarctica of ∼10^2^ cells mL^−1^, which is perhaps one of the most relevant environmental analogues for Europa that can be found on Earth (Christner *et al.,* 2006). Whether or not cell densities at the ice-water interface are conserved and represented via transport to the surface depends on the variety of potential mechanisms that could deliver basal ice to the surface (see, *e.g.,* Collins and Nimmo, 2009). Prospects for life detection on ice-covered ocean worlds could be significantly enhanced if the ice-water interface harbors enough redox chemistry to sustain sub-ice shell biofilms (McKay *et al.,* 2008, 2014; Hand *et al.,* 2009, 2017; Shock and Boyd, 2015; Lorenz, 2016; Nimmo and Pappalardo, 2016; Sparks *et al.,* 2016). Detection techniques are itemized in Hand *et al.* (2017) where, for example, atomic force microscopy as well as deep-UV Raman spectroscopy are considered (Sivakumar *et al.,* 2015; Abbey *et al.,* 2017). It is notable that atomic force microscopes have already been flown on the Phoenix and Rosetta missions (Pike *et al.,* 2011; Bentley *et al.,* 2016).

## 4. Discussion

That we have not considered methanogenesis as a possible metabolism needs explanation. Of course, methanogens on Earth do need to expel excess electrons, but these are generally borne away physically as methane gas itself, although some do appear to reduce Fe(III) at the same time (Vargas *et al.,* 1998). Be that as it may, it is doubtful if methanogens would have made up a significant portion of the microbial population on Europa or other icy moons given the high concentrations of abiotic methane likely emanating from the reduced mantles (Gaidos *et al.,* 1999, 2009; McDermott *et al.,* 2015; *cf.* Waite *et al.,* 2017). And they were certainly unlikely to have emerged at an early stage in the emergence of life on such a moon, as conditions would have strongly favored methanotrophy instead, with sulfate, sulfur, and/or ferric iron as oxidants (Nitschke and Russell, 2013; McGlynn, 2017; Russell and Nitschke, 2017; *cf.* Milucka *et al.,* 2012; Egger *et al.,* 2017).

## 5. Conclusions

The dynamic communities comprising any sub-ice-shell biosphere could constitute a habitable zone, manifested as microbial mats concentrated around oxidant-rich ice-shell sites at the down-welling regions of thermally unstable regions at the ice-water interface (Goodman *et al.,* 2004). An expectation of this physiochemically dynamic freeze-thaw scenario is that cells would be periodically trapped in the ice, and these communities brought to the surface in a cryogenic state along with their ambient environmental molecules (Showman and Han, 2005; Porco *et al.,* 2006; Spencer *et al.,* 2006; Waite *et al.,* 2006, 2017; Spitale and Porco, 2007; McKay *et al.,* 2008; Roth *et al.* 2014; Nadeau *et al.,* 2016; Vance *et al.,* 2016; Hand *et al.,* 2017). Thus, along with the standard methods for flying through possible plumes, and deploying landers, future missions could eventually use robotic capabilities designed for exploring the ice-water interface (Berisford *et al.,* 2012; Ananthaswamy, 2013).

Future missions to search for life on Europa (Hand *et al.,* 2017), a young Enceladus (McKay *et al.,* 2014; Ćuk *et al.,* 2016), and other ocean words will be tasked with seeing if life emerged afresh elsewhere, independently from Earth where Snowball events were relatively short-lived (Harland, 1964; Kirschvink, 1992; Hoffman and Schrag, 2002; Parkinson *et al.,* 2008; Blanc *et al.,* 2016; Barge and White, 2017). A difference may be that the putative chemostatic biospheres on extraterrestrial, and even extrasolar, icy worlds may, for millions or billions of years, have been dynamically stable long after the emergence of life thereon, supported by serpentinization—life's mother engine (Russell *et al.,* 1989, 2010; Branscomb and Russell, 2013).
